# *N*-Heterocyclic Carbene-Catalyzed Random Copolymerization of *N*-Carboxyanhydrides of α-Amino Acids

**DOI:** 10.3390/polym13213674

**Published:** 2021-10-25

**Authors:** Kuen Hee Eom, Seokhyeon Baek, Il Kim

**Affiliations:** Department of Polymer Science and Engineering, Pusan National University, Busandaehag-ro 63-2, Geumjeong-gu, Busan 46241, Korea; reimreim@pusan.ac.kr (K.H.E.); skb1130@pusan.ac.kr (S.B.)

**Keywords:** amino acids, *N*-carboxyanhydrides, catalysts, copolymerization, *N*-heterocyclic carbene, polypeptides, random copolypeptides, reactivity ratio

## Abstract

Synthetic polypeptides prepared from *N*-carboxyanhydrides (NCAs) of α-amino acids are useful for elucidating the relationship between the primary structure of natural peptides and their immunogenicity. In this study, complex copolypeptide sequences were prepared using a recently developed technique; specifically, the random copolymerization of l-alanine NCA with NCAs of l-glutamic acid 5-benzylester (Bn-Glu NCA), *S*-benzyl-cysteine (Bn-Cys NCA), *O*-benzyl-l-serine (Bn-Ser NCA), and l-phenylalanine (Phe NCA) was performed using *N*-heterocyclic carbene (NHC) catalysts. The NHC-initiated Ala NCA/Bn-Glu NCA and Ala NCA/Bn-Cys NCA copolymerization reactions achieved 90% conversion within 30 min. The reactivity ratio values estimated using the Kelen and Tüdos method show that poly(Bn-Glu-*co*-Ala) and poly(Bn-Cys-*co*-Ala) have random repeating units with rich alternating sequences, whereas poly(Bn-Ser-*co*-Ala) and poly(Phe-*co*-Ala) contain a larger proportion of Ala-repeating units than Bn-Ser and Phe in random placement.

## 1. Introduction

Synthetic peptide-based polymers are a class of promising materials that exhibits interesting self-assembly behaviors and shows promise for applications in drug release, gene delivery, tissue engineering, and regenerative medicine [1,2,3,4,5] as well as metal-free polypeptide-based battery applications [6]. In recent decades, improved methods in the ring-opening polymerization (ROP) of α-amino acid-*N*-carboxyanhydrides (NCAs) have enabled the preparation of increasingly complex copolypeptide sequences having controlled molecular weights (MWs) that display properties far superior to those of ill-defined homo- and copolypeptides, which are difficult to prepare using conventional methods owing to the slow polymerization rate (typically requiring hours to days to finish) and suffering from various side reactions, including chain termination, chain transfer, and water-induced NCA degradation [5,7,8,9,10,11,12,13]. Fortunately, well-defined polypeptides with complex structures, high MWs, and unique block sequences are now accessible in an efficient manner even in the presence of moisture.

Although the recent developments in the polymerization of NCAs have improved the efficiency and simplicity of the techniques for the preparation of polypeptides and enabled the preparation of polymers that were previously difficult to prepare using conventional methods, studies of the random copolymerization process in terms of the reactivity ratio of the various NCA monomers are surprisingly scarce [14,15,16,17,18,19,20,21,22,23,24], especially for recently developed ROP systems. In most cases, for copolypeptides prepared via NCA copolymerization, it is assumed that a random sequence distribution is achieved [25]. Based on conventional copolymerization experiments using a range of NCAs and primary amines as initiators, the reactivity order decreases as follows: Gly NCA >Ala NCA > γ-BzGlu NCA > Leu NCA > Val NCA.

**Scheme 1:**
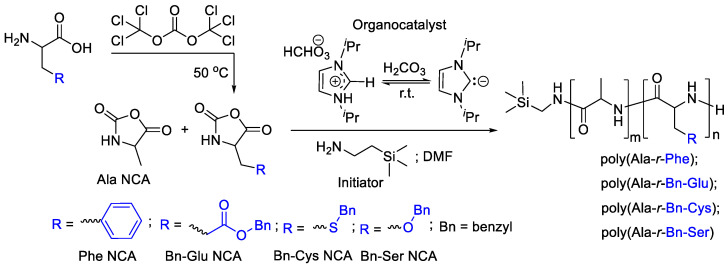
Organocatalyzed copolymerization of Ala NCA with four characteristic amino acid NCAs: Phe NCA, Bn-Glu NCA, Bn-Cys NCA, and Bn-Ser NCA, which were chosen from four distinct amino acid categories.

Recently, we developed an organocatalyst system that shows fast polymerization kinetics that outpace common side reactions, thus enabling the preparation of well-defined polypeptides [12]. More specifically, we found that air-stable *N*-heterocyclic carbene (NHC) precursors ([RNHC(H)]^+^[HCO_3_]^−^) [26] can serve as efficient catalysts for the zwitterionic ROP of NCA monomers, thus yielding linear/cyclic polypeptides with narrow polydispersities and adjustable MWs. Considering that random copolymerization is a good tool to produce copolypeptides on a large scale as well as to design diverse structures, in this study, the random copolymerization of Ala NCA with NCAs chosen from four distinct categories was carried out. The four classes of amino acids include those having a negatively charged hydrophilic side chain (Bn-Glu), polar but uncharged side chain (Bn-Ser), a special side chain (Bn-Cys), and nonpolar hydrophobic chain (Phe). Scheme 1 summarizes the copolymerization systems used in this study. Intriguingly, the reactivity ratios estimated in this study show dramatic differences based on the monomer and comonomer pairs. In particular, the resultant copolypeptides showed statistical and alternating repeating units rather than blocky units.

## 2. Materials and Methods

All syntheses and polymerizations were performed in a glove box or with Schlenk techniques under a nitrogen atmosphere. Anhydrous dimethylformamide (DMF) was purchased from Thermo Fisher Scientific (Seoul, Korea) and used without further purification. Tetrahydrofuran (THF), acetone, and methanol were purified by treatment with calcium disulfide under nitrogen. 1,3-Diisopropylimidazolium chloride was purchased from Sigma–Aldrich and stored in a glove box. (Trimethylsilyl)methylamine, l-alanine, *S*-benzyl-cysteine, *O*-benzyl-l-serine, l-glutamic acid 5-benzylester, and l-phenylalanine were purchased from Thermo Fisher Scientific and used as received. All other chemicals, such as triphosgene and trifluoroacetic acid (TFA), were purchased from Sigma-Aldrich and used as received.

### 2.1. Synthesis

The 1,3-diisopropylimidazolium hydrogen carbonate ([iPrNHC(H)]^+^[HCO_3_]^−^) catalyst precursor was synthesized by reacting 1,3-diisopropylimidazolium chloride with KHCO_3_ following a reported procedure [12,26].

For the synthesis of (*S*)-γ-benzyl-l-glutamate-*N*-carboxyanhydride (Bn-Glu NCA), l-glutamic acid 5-benzyl ester (H–Glu(OBzl)–OH) (2.37 g, 10 mmol) was suspended in 40 mL of freshly distilled THF and fed to a Schlenk flask (250 mL) fitted with a N_2_ inlet and a rubber septum at 40 °C. Then, triphosgene (1.49 g, 5 mmol) dissolved in THF (10 mL) was slowly added (1 drop/second) using a syringe pump. Once the suspension had become clear (within 2 h), the reaction mixture was cooled to room temperature, purged with N_2_ to remove unreacted phosgene and HCl, and concentrated under vacuum. The crude product was recrystallized using excess anhydrous hexane and THF three times and dried under vacuum to afford white crystals. Yield: 1.96 g (73%). ^1^H NMR (fully deuterated dimethyl sulfoxide (DMSO-*d*_6_), 400 MHz): δ (ppm) = 1.92 (m, 1H, *γ*-CH), 2.02(m, 1H, *γ*-CH), 2.52 (t, 2H, *β*-CH_2_), 4.49 (dd, 1H, *α*-CH), 5.10 (s, 2H, –CH_2_Ar), 7.35 (m, 5H, ArH), and 9.11 (s, 1H, –NH–). ^13^C NMR (DMSO-*d*_6_, 100 MHz): δ (ppm) = 27.1 (*β*-CH2), 29.7 (*γ*-CH2), 57.0 (*α*-CH2), 57.0 (–CH_2_Ar), 128.7, 128.8, 129.2, 136.7 (ArH), 152.3 (–NHC(O)O–), 172.0 (–C(O)OCH_2_–), and 174.2 (–C(O)OC(O)–).

Ala NCA was synthesized by reacting l-alanine with triphosgene following the same procedure as that used for the synthesis of Bn-Glu NCA except that the reaction temperature was increased to 55 °C. ^1^H NMR (CDCl_3_, 400 MHz): δ (ppm) = 1.58 (d, 3H, –CH_3_), 4.42 (q, 1H, –CH–), and 6.30 (s, 1H, –NH–). ^13^C NMR (DMSO-*d*_6_, 100 MHz): δ (ppm) = 19.3 (–CH_3_), 59.8 (–CH–), 153.1 (–NHC(O)O–), and 171.9 (–C(O)OC(O)–).

Bn-Cys NCA was synthesized by reacting *S*-benzyl-cysteine with triphosgene following the same procedure as that used for the synthesis of Bn-Glu NCA. ^1^H NMR (CDCl_3_, 400 MHz): δ (ppm) = 2.80 (m, 2H, –CH_2_–), 3.77 (s, 2H, –CH_2_Ar), 4.33 (q, 1H, –CH–), 6.44 (s, 1H, –NH–), and 7.31 (m, 5H, ArH). ^13^C NMR (CDCl_3_, 100 MHz): δ (ppm) = 2.6 (–CH_2_–), 37.0 (–CH_2_Ar), 57.7 (–CH–), 127.7, 128.1, 128.9, 137.0 (ArH), 152.1 (–NHC(O)O–), and 175.1 (–C(O)OC(O)–).

Phe NCA was synthesized by reacting l-phenylalanine with triphosgene following the same procedure as that used for the synthesis of Bn-Glu NCA. ^1^H NMR (DMSO-*d*_6_, 400 MHz): δ (ppm) = 3.07 (br, 2H, –CH_2_–), 4.81 (m, 1H, –CH–), 7.25 (m, 5H, ArH), and 9.10 (s, 1H, –NH–). ^13^C NMR (DMSO-*d*_6_, 100 MHz): δ (ppm) = 36.5 (–CH_2_–), 63.6 (–CH–), 125.4, 127.7, 128.6, 136.6 (ArH), 149.0 (–NHC(O)O–), and 171.7 (–C(O)OC(O)–).

Bn-Ser NCA was synthesized by reacting *O*-benzyl-l-serine with triphosgene following the same procedure as that used for the synthesis of Bn-Glu NCA. ^1^H NMR (CDCl_3_, 400 MHz): δ (ppm) = 2.80 (m, 2H, –CH_2_–), 3.77 (s, 2H, –CH_2_Ar), 4.33 (q, 1H, –CH–), 6.44 (s, 1H, –NH–), and 7.31 (m, 5H, ArH). ^13^C NMR (CDCl_3_, 100 MHz): δ (ppm) = 2.6 (–CH_2_–), 37.0 (–CH_2_Ar), 57.7 (–CH–), 127.7, 128.1, 128.9, 137.0 (ArH), 152.1 (–NHC(O)O–), and 175.1 (–C(O)OC(O)–).

### 2.2. Random Copolymerization

In a typical copolymerization of Ala NCA and Bn-Glu NCA having the same feed composition, Ala NCA (57.55 mg, 0.5 mmol) and Bn-Glu NCA (131.63 mg, 0.5 mmol) were dissolved in 5 mL of DMF in a Schlenk flask. Molecular sieves and (trimethylsilyl)methyl amine initiator (4.13 mg, 2 × 10^−^^5^ mol) were then introduced into the solution, and the ([*i*PrNHC(H)]^+^[HCO_3_]^−^) catalyst precursor/DMF stock solution (500 µL, 2 × 10^−^^5^ mol, 0.04 M) was then added to the reaction mixture using a syringe. The reaction mixture was stirred for 1 h at 25 °C. The progress of the polymerization reaction was traced by NMR spectroscopy, and the NCA conversion was determined by comparing the NCA concentration in the solution with that at time (*t*) = 0. After completion of the reaction (as monitored by Fourier-transform infrared (FT-IR) spectroscopy), the solution was precipitated in excess methanol. After sonication and centrifugation twice to remove the unreacted monomers, the obtained white solid was dried under vacuum. Details of the conditions used for the copolymerization of Ala NCA with Bn-Glu NCA, Bn-Cys NCA, Bn-Ser NCA, and Phe NCA are shown in Table 1. Note that the reactivity ratios were estimated based on the samples collected within 5 min of copolymerization to minimize compositional heterogeneity by limiting the monomer conversion to below 5%.

### 2.3. Characterization

^1^H and ^13^C NMR spectra were recorded on a Varian Unity 400 MHz spectrometer, and the chemical shifts are reported in parts per million downfield from tetramethylsilane and referenced to the residual solvent peak. 2D-NMR (heteronuclear single quantum coherence (HSQC)) spectra were recorded on a 500 MHz Prodigy NMR AVANCE NEO 500.

## 3. Results and Discussion

### 3.1. [iPrNHC(H)][HCO_3_]-Mediated Copolymerization of Ala NCA with Bn-Glu NCA, Bn-Cys NCA, Bn-Ser NCA, and Phe NCA

A general and highly straightforward strategy for the synthesis of both linear and cyclic polypeptides that employs the NHC-mediated living polymerization of NCA was recently reported by our group [12]. The fast and controlled polymerization of a variety of NCAs, which results in high conversions within a few minutes under ambient conditions, is easily achieved by tuning the type of initiator and organocatalyst. In addition, the use of living polymerization results in highly uniform block copolymers by the sequential addition of NCA monomers. This organocatalyzed polymerization technique provides a potent protocol for the synthesis of polypeptide topologies having ever-increasing complexity, thus enabling the mimicry of natural nanostructures.

**Figure 1:**
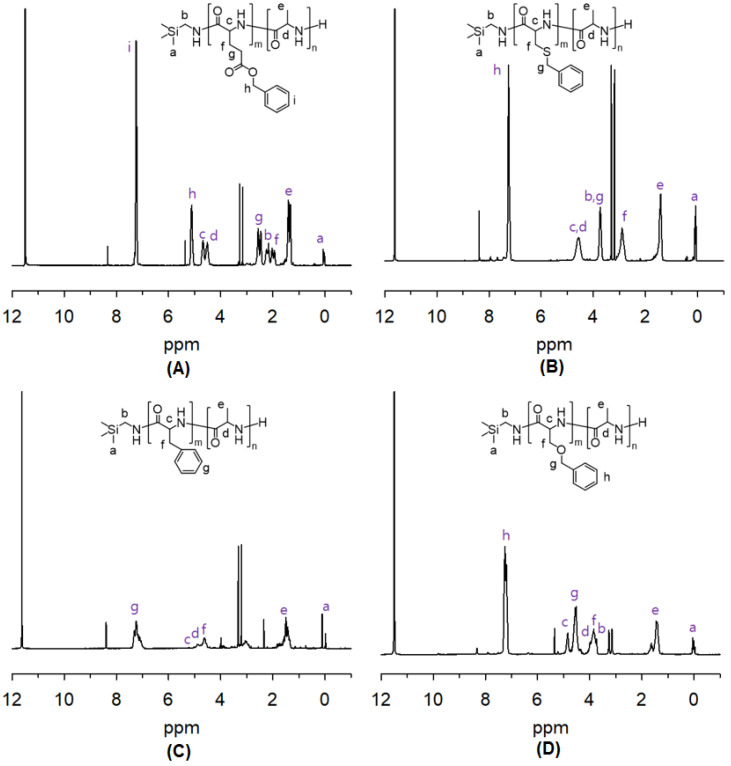
^1^H NMR spectra of (**A**) poly(Ala-*co*-Bn-Glu), (**B**) poly(Ala-*co*-Bn-Cys), (**C**) poly(Ala-*co*-Phe), and (**D**) poly(Ala-*co*-Bn-Ser) measured in TFA-*d*.

As a continuation of this work, the random copolymerization of Ala NCA with Bn-Glu NCA, Bn-Cys NCA, Bn-Ser NCA, and Phe NCA, which were chosen from four representative categories of amino acids, were performed. All copolymerization reactions reached high conversion within 30 min, demonstrating the powerful catalytic activity of the NHC catalysts in the presence of the (trimethylsilyl)methylamine initiator. Figure 1 shows the ^1^H and ^13^C NMR spectra of poly(Ala-*co*-Bn-Glu), poly(Ala-*co*-Bn-Cys), poly(Ala-*co*-Bn-Ser), and poly(Ala-*co*-Phe) prepared at the same feed composition (*f*_1_/ *f*_2_ = 1) for 1 h. Here, *f*_1_ represents the feed composition of Bn-Glu NCA, Bn-Cys NCA, Bn-Ser NCA, or Phe NCA, and *f*_2_ represents the feed composition of Ala NCA. The copolymerization of Ala NCA with Bn-Glu NCA and Cys NCA reached more than 90% conversion within 30 min, and the copolymerization of Ala NCA with Bn-Ser NCA and Phe NCA reached more than 30% conversion within 10 min. However, subsequently, the reaction mixture was changed to a heterogeneous system because of the poor solubility of the resultant copolymers. Specifically, poly(Ala-*co*-Bn-Ser) showed the worst solubility in deuterated TFA (TFA-*d*), making it difficult to collect ^13^C NMR spectra. The formation of the copolymers was clearly confirmed by the presence of the methyl peaks corresponding to the initiator at 0.22 ppm, the pendant methyl peak in the Ala unit at around 1.5 ppm, and the benzylic protecting group of each comonomer at around 7.5 ppm. Accordingly, the relative composition of Ala in the comonomer could be estimated by the integration of the representative signals of each unit.

The copolymerization performed at *f*_1_/ *f*_2_ = 1 ([M]_0_/[I]_0_ = 25/1; [M]_0_ and [I]_0_ are the initial monomer (and comonomer) and initiator concentrations) resulted in different copolymer compositions (*F*_1_ and *F*_2_): *F*_1_/ *F*_2_ = 0.67, 0.54, 0.22, and 0.19 for poly(Ala-*co*-Bn-Glu), poly(Ala-*co*-Bn-Cys), poly(Ala-*co*-Phe), and poly(Ala-*co*-Bn-Ser), respectively, having MWs of 4900, 2800, 2900, and 3100, respectively. The ^13^C NMR spectra (Figure S1, Supplementary Material) and HSQC spectra (Figure S2) of the copolymers were also collected. Assignments made on the figures support the successful formation of random copolymers. Note that the collection of data from poly(Ala-*co*-Bn-Ser) was unsuccessful because of the previously mentioned solubility problem.

### 3.2. Reactivity Ratios for the Copolymerization of Ala NCA with Bn-Glu NCA, Bn-Cys NCA, Bn-Ser NCA, and Phe NCA

**Figure 2:**
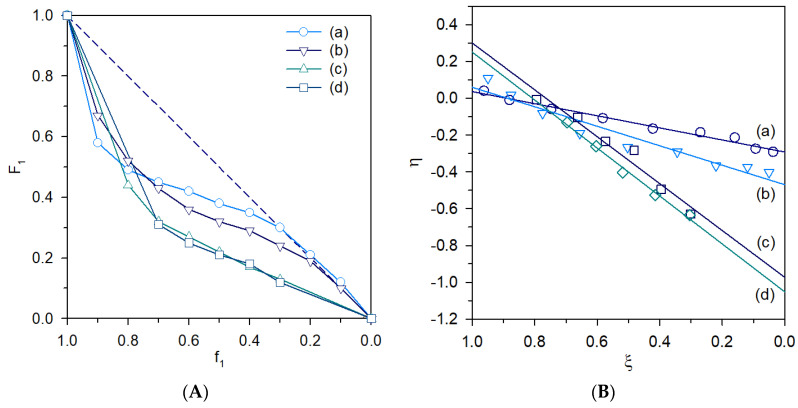
(**A**) Dependence of the copolymer composition, *F*_1_, on the initial comonomer feed composition, *f*_1_, and (**B**) Kelen–Tüdos plots for the determination of the reactivity ratios for (a) Bn-Glu NCA and Ala NCA, (b) Bn-Cys NCA and Ala NCA, (c) Bn-Ser NCA and Ala NCA, and (d) Phe NCA and Ala NCA copolymerization systems, where Ala NCA is monomer 2 in all cases.

Four comonomers, Bn-Glu NCA, Bn-Cys NCA, Bn-Ser NCA, and Phe NCA, were selected for reaction with Ala NCA. These selections were made based on the different characteristics of these amino acids, which suggest that their copolymerization may be affected by their different reactivities and result, for example, in the preferential inclusion of the more reactive monomer in the copolymer chain. Because the more reactive monomer is preferentially incorporated into the copolymer, the monomer ratio in the feed rapidly changes, resulting in significant heterogeneity in the copolymer composition with increasing conversion. To cope with this problem, we attempted to minimize the compositional heterogeneity by limiting the synthesis to relatively low conversions by performing copolymerization for 5 min. The molar ratio of Ala NCA in the feed was set over a wide range (0.1–0.9) so that the content of Ala NCA and each comonomer in the copolymer would be evenly distributed over a reasonably broad range. Table 1 summarizes the feed ratio, copolymer composition, and molecular weight of the copolymers. The compositions of the copolymers were determined by integrating the peaks corresponding to Ala NCA and the comonomer units in the ^1^H NMR spectra shown in Figure S3. Figure 2A shows the copolymer composition diagrams for the copolymerization of Ala NCA with Bn-Glu NCA, Bn-Cys NCA, Bn-Ser NCA, and Phe NCA.

To determine the reactivity ratios of Ala NCA (*r*_2_) and four comonomers (*r*_1_) based on the data collected from Figure 2A, we employed the widely used linear graphical method developed by Kelen and Tüdos [27]. The Kelen and Tüdos method was developed assuming that, under extreme experimental conditions, including largely mismatched monomer reactivities and a very low concentration of one of the monomers, the experimental data may not be unequally weighted, as in the method developed by Fineman and Ross [28]. Kelen and Tüdos used Equation (1).

(1) $$\eta =\left(r_{1}+\frac{r_{2}}{\alpha }\right)\xi -\frac{r_{2}}{a}$$

Here, η = *G*/(*α* + *H*), ξ = *H*/(*α* + *H*), G = *X*(*Y* − 1)/Y, *H* = *X*^2^/*Y*, *X* = *f*_1_/*f*_2_, and *Y* = *F*_1_/*F*_2_. Here, α is an arbitrary positive constant. A feasible choice is α = (*H*_m_ ∙ *H*_M_)^1/2^, where *H*_m_ and *H*_M_ are the lowest and highest values of *H*, respectively.

The linear extrapolation plots obtained using the Kelen and Tüdos method are shown in Figure 2B. Note that, even using Kelen and Tüdos refinement, there are statistical limitations inherent to the linearization method. Specifically, the independent variable in any form of the linear equation is not really independent, and the dependent variable does not have a constant variance because Equation (1) assumes that there is no variation in the feed composition during the copolymerization, but this is not true. Although we held the conversion as low as possible, there must be a measurable change in the feed composition. Kelen and Tudos modified Equation (1) to transform experimental data even at high conversions [29]. In this method, an average monomer composition is assigned to the corresponding experimental average copolymer composition. The proposed approximation extends the use of prior Kelen and Tüdos method developed for low conversions. This extended method redefined *G* and *H* into *G* = (*Y* − 1)/*z* and *H* = *Y*/*z*^2^, where *z* = log_10_(1 − *τ*_1_)/log_10_(1 − *τ*_2_); *τ*_2_ = (*w*/100)(*μ* + *X*)/(*μ* + *Y*); *τ*_1_ = *τ*_2_(*Y*/*X*); *μ* = (MW of monomer 2)/(MW of monomer 1), and *w* is the weight percent conversion of total monomers. Figure S4 shows the extended Kelen and Tüdos plots for the four copolymerization systems, which are similar to those in Figure 2B.

**Figure 3:**
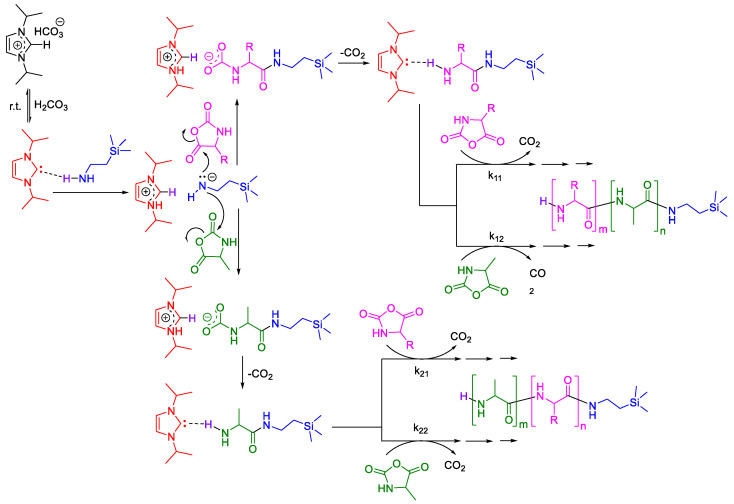
Proposed mechanism of NHC-initiated random copolymerization of Ala NCA with Bn-Glu NCA, Bn-Cys NCA, Bn-Ser NCA, and Phe NCA.

The reactivity ratios of the Bn-Glu NCA (*r_*1*, Bn-Glu NCA_*) and Ala NCA (*r*_2_), Bn-Cys NCA (*r_*1*, Bn-Cys NCA_*) and Ala NCA (*r*_2_), Bn-Ser NCA (*r_*1*, Bn-Ser NCA_*) and Ala NCA (*r*_2_), and Phe NCA (*r_*1*, Phe NCA_*) and Ala NCA (*r*_2_) copolymerization reactions were 0.04 and 0.65, 0.06 and 0.98, 0.25 and 4.43, and 0.30 and 4.17, respectively, and the *r*_1_∙*r*_2_ values of the four copolymerization systems were 0.026, 0.059, 1.108, and 1.251, respectively. The reactivity ratios estimated based on extended Kelen and Tüdos method were 0.03 and 0.63, 0.06 and 0.92, 0.33 and 4.36, and 0.26 and 4.56 for the Bn-Glu NCA (*r_*1*, Bn-Glu NCA_*) and Ala NCA (*r*_2_), Bn-Cys NCA (*r_*1*, Bn-Cys NCA_*) and Ala NCA (*r*_2_), Bn-Ser NCA (*r_*1*, Bn-Ser NCA_*) and Ala NCA (*r*_2_), and Phe NCA (*r_*1*, Phe NCA_*) and Ala NCA (*r*_2_) copolymerizations, respectively. Both *r*_1_ and *r*_2_ values were less than one for the Bn-Glu NCA/Ala NCA and Bn-Cys NCA/Ala NCA copolymerization reactions. Specifically, the reactivity ratios of Bn-Glu NCA (0.04) and Bn-Cys NCA (0.06) in these copolymerization systems are very small, indicating that the probability of the addition of another Bn-Glu NCA unit after the addition of Bn-Glu NCA units is very low (see Figure 3). This is the case for Bn-Cys NCA/Ala NCA copolymerization. Thus, the behavior of these comonomer systems lies between those of ideal and alternating copolymerization. Because the *r*_1_•*r*_2_ values are very small, 0.026 and 0.059, respectively, there is a tendency away from ideal copolymerization behavior.

In the case of the Bn-Ser NCA/Ala NCA and Phe NCA/Ala NCA copolymerization reactions, for which the two monomer reactivity ratios are different, that is, *r*_1_ < 1 and *r*_2_ > 1, Ala NCA is more reactive than Bn-Ser NCA and Phe NCA toward the propagating species. Thus, the copolymer contained a larger proportion of Ala NCA than Bn-Ser NCA or Phe NCA in random placement. There have been no reports on the copolymerization systems investigated in this study; however, as summarized in Table 2, the reactivity ratios of the amine-initiated copolymerization systems, such as Ala NCA/Leu NCA [29], Ala NCA/Val NCA [16], Ala NCA/*O*-Acetyl Tyr NCA [30], and Ala NCA/Trp NCA [21], indicate that the resultant copolymers have similar structures to those of the NHC-catalyzed Ala NCA/Bn-Ser NCA and Ala NCA/Phe NCA copolymers prepared in this study, whereas the reactivity ratios of Ala NCA/Gly NCA [29] are less than unity, such as the Ala NCA/Bn-Glu NCA and Ala NCA/Bn-Cys NCA copolymerization systems.

## 4. Conclusions

As an initial trial to design complex copolypeptides, the NHC-initiated copolymerization reactions of Ala NCA with Bn-Glu NCA, Bn-Cys NCA, Be-Ser NCA, and Phe NCA, which were chosen from four different categories of amino acids, showed high reactivity under mild conditions. In particular, the Ala NCA/Bn-Glu NCA and Ala NCA/Bn-Cys NCA copolymerization systems showed very fast kinetics, yielding 90% conversion at 25 °C within 30 min. To estimate the reactivity ratios, a series of copolymerization reactions were performed for 5 min to minimize the compositional heterogeneity by limiting the reaction to only relatively low conversions. In contrast, the molar ratio of Ala NCA to comonomer in the feed was set in a wide range from 0.1 to 0.9 so that the content of Ala NCA and each comonomer in the copolymer would be evenly distributed over a reasonably broad range.

The reactivity ratios for the copolymerization of Bn-Glu NCA (*r_*1*, Bn-Glu NCA_*)/Ala NCA (*r*_2_), Bn-Cys NCA (*r_*1*, Bn-Cys NCA_*)/Ala NCA (*r*_2_), Bn-Ser NCA (*r_*1*, Bn-Ser NCA_*)/Ala NCA (*r*_2_), and Phe NCA (*r_*1*, Phe NCA_*)/Ala NCA (*r*_2_) estimated using the Kelen and Tüdos method are 0.04 and 0.65, 0.06 and 0.98, 0.25 and 4.43, and 0.30 and 4.17, respectively. The reactivity ratios and their product (*r*_1_*∙r*_2_) values demonstrate that poly(Bn-Glu-*co*-Ala) and poly(Bn-Cys-*co*-Ala) have random repeating units with rich alternating sequences, whereas poly(Bn-Ser-*co*-Ala) and poly(Phe-*co*-Ala) contain a larger proportion of Ala-repeating units than Bn-Ser and Phe with random placement. The reactivity ratio data collected in this study can be used as a basis for designing copolypeptides having complex structures mimicking those of natural peptide sequences. Well-defined homo- and copolypeptides can be innovative materials in biomedical applications, including controlled drug/gene delivery systems, tissue engineering, molecular imaging, and diagnostics.

## Figures and Tables

**Table 1:** Results of copolymerizations of Ala NCA with Bn-Glu NCA, Bn-Cys NCA, Phe NCA, and Be-Ser NCA.

Entry	NCA1	NCA2	Composition in Feed		Composition in Polymer ^1^		Time (min)	*w*^2^ (%)	*M*_n_ ^1^ (kg·mol^−1^)
			NCA1	NCA2	NCA1	NCA2			
**1**	Bn-Glu	Ala	0.9	0.1	0.58	0.42	3	2.9	6200
**2**	Bn-Glu	Ala	0.8	0.2	0.49	0.51	3	2.9	6000
**3**	Bn-Glu	Ala	0.7	0.3	0.45	0.55	3	3.1	5400
**4**	Bn-Glu	Ala	0.6	0.4	0.42	0.58	3	3.3	4900
**5**	Bn-Glu	Ala	0.5	0.5	0.38	0.62	3	3.3	4800
**6**	Bn-Glu	Ala	0.4	0.6	0.35	0.65	3	3.4	4500
**7**	Bn-Glu	Ala	0.3	0.7	0.30	0.70	3	3.6	3200
**8**	Bn-Glu	Ala	0.2	0.8	0.21	0.79	3	3.5	2600
**9**	Bn-Glu	Ala	0.1	0.9	0.12	0.88	3	3.9	1700
**10**	Bn-Cys	Ala	0.9	0.1	0.67	0.33	3	2.3	4000
**11**	Bn-Cys	Ala	0.8	0.2	0.52	0.48	3	2.3	3300
**12**	Bn-Cys	Ala	0.7	0.3	0.43	0.57	3	2.5	3100
**13**	Bn-Cys	Ala	0.6	0.4	0.36	0.64	3	2.4	2500
**14**	Bn-Cys	Ala	0.5	0.5	0.32	0.68	3	2.3	2500
**15**	Bn-Cys	Ala	0.4	0.6	0.29	0.71	3	2.6	2400
**16**	Bn-Cys	Ala	0.3	0.7	0.24	0.76	3	2.8	2300
**17**	Bn-Cys	Ala	0.2	0.8	0.18	0.82	3	2.8	2200
**18**	Bn-Cys	Ala	0.1	0.9	0.10	0.90	3	3.3	4000
**19**	Phe	Ala	0.8	0.2	0.49	0.51	5	1.5	2600
**20**	Phe	Ala	0.7	0.3	0.39	0.61	5	1.5	2600
**21**	Phe	Ala	0.6	0.4	0.28	0.72	5	1.5	2400
**22**	Phe	Ala	0.5	0.5	0.20	0.80	5	1.7	2200
**23**	Phe	Ala	0.4	0.6	0.14	0.86	5	1.6	2200
**24**	Phe	Ala	0.3	0.7	0.09	0.91	5	1.7	1700
**25**	Bn-Ser	Ala	0.7	0.3	0.36	0.64	5	1.2	4400
**26**	Bn-Ser	Ala	0.6	0.4	0.26	0.74	5	1.5	4100
**27**	Bn-Ser	Ala	0.5	0.5	0.18	0.82	5	1.5	2900
**28**	Bn-Ser	Ala	0.4	0.6	0.13	0.87	5	1.7	2700
**29**	Bn-Ser	Ala	0.3	0.7	0.09	0.91	5	1.6	2400

^1^ Determined by ^1^H NMR spectroscopy. ^2^ Weight percent conversion of total monomers determined by ^1^H NMR spectra shown in Figure S3.

**Table 2:** Reactivity ratios of Ala NCA for copolymerization with NCAs of some α-amino acids.

Monomer 1 ^1^	*r*_1_	*r*_2_	Solvent	Catalyst	Conditions	Reference
Leu NCA	0.35	1.7	Acetonitrile	No catalyst	30 °C, 2 h	[16]
Gly NCA	0.46–0.60	0.21–0.23	Acetonitrile	No catalyst	30 °C, 2 h	[16]
Val NCA	0.09–0.13	2.4–2.6	Acetonitrile	No catalyst	30 °C, 2 h	[16]
O-Acetyl Tyr NCA	0.12	2.7	Ethyl acetate	No catalyst	25 °C, 2 h	[30]
Trp NCA	0.2	10.5	Dioxane	No catalyst	30 °C, 15 d	[21]
Bn-Glu NCA	0.04	0.65	DMF	NHC	25 °C, 5 min	This study
Bn-Cys NCA	0.06	0.98	DMF	NHC	25 °C, 5 min	This study
Bn-Ser NCA	0,25	4.43	DMF	NHC	25 °C, 5 min	This study
Phe NCA	0.30	4.17	DMF	NHC	25 °C, 5 min	This study

^1^ In all cases, monomer 2 is Ala NCA.

## Data Availability

The data presented in this study are available on request from the corresponding author.

## Supplementary Materials

The following are available online at https://www.mdpi.com/article/10.3390/polym13213674/s1, Figure S1: ^13^C NMR spectra of (a) poly(Ala-*co*-Bn-Glu), (b) poly(Ala-*co*-Bn-Cys), and (c) poly(Ala-*co*-Phe) measured in TFA-*d*; Figure S2: HSQC spectra of (a) poly(Ala-*co*-Bn-Glu), (b) poly(Ala-*co*-Bn-Cys), and (c) poly(Ala-*co*-Phe) measured in TFA-*d*; Figure S3: ^1^H NMR spectra of (a) poly(Ala-*co*-Bn-Glu), (b) poly(Ala-*co*-Bn-Cys), (c) poly(Ala-*co*-Phe), and (d) poly(Ala-*co*-Bn-Ser) after 5 min of copolymerization over a wide range of relative compositions. Some of the copolymer samples could not be analyzed because of their low solubility in TFA-*d*; Figure S4: Extended Kelen–Tüdos plots for the determination of the reactivity ratios for (a) Bn-Glu NCA and Ala NCA, (b) Bn-Cys NCA and Ala NCA, (c) Bn-Ser NCA and Ala NCA, and (d) Phe NCA and Ala NCA copolymerization systems, where Ala NCA is monomer 2 in all cases.
