# Supplementary material for: N-Heterocyclic Carbene-Catalyzed Random Copolymerization of N-Carboxyanhydrides of α-Amino Acids
Source: Polymers (Basel). 2021 Oct 25;13(21):3674. doi: 10.3390/polym13213674 (PMC8586994; doi:10.3390/polym13213674)
Supplement: Supplementary file 1 [file polymers-13-03674-s001.zip › polymers-1429758-supplementary.pdf]

polymers

MDPI

Supplementary Materials: *N*-Heterocyclic Carbene-catalyzed Random Copolymerization of *N*-carboxyanhydrides of α-amino Acids

Kuen Hee Eom, Seokhyeon Baek and Il Kim*

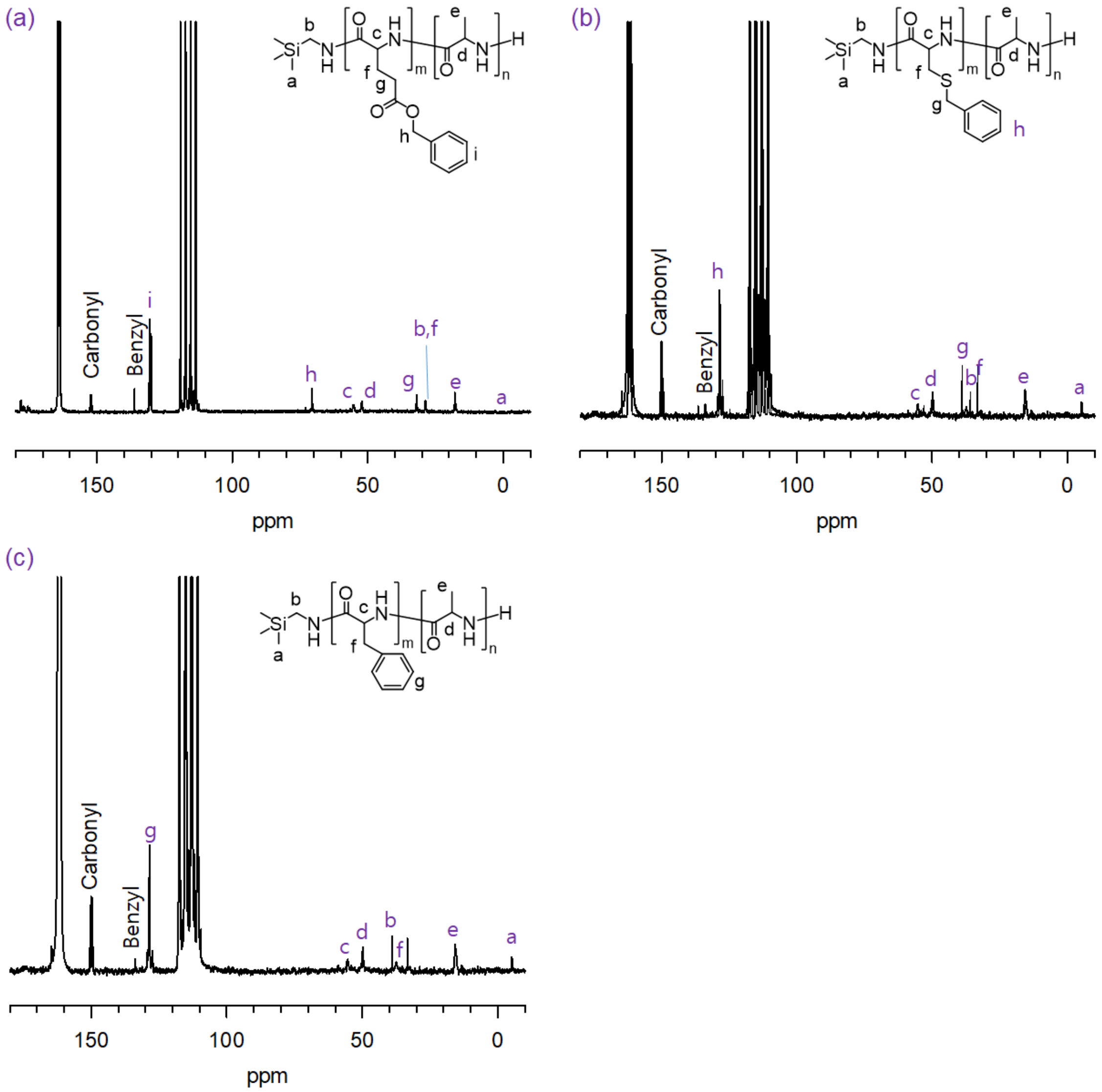

Figure S1. ^{13}C NMR spectra of (**a**) poly(Ala-*co*-Bn-Glu), (**b**) poly(Ala-*co*-Bn-Cys), and (**c**) poly(Ala-*co*-Phe) measured in trifluoroacetic acid (TFA-d).

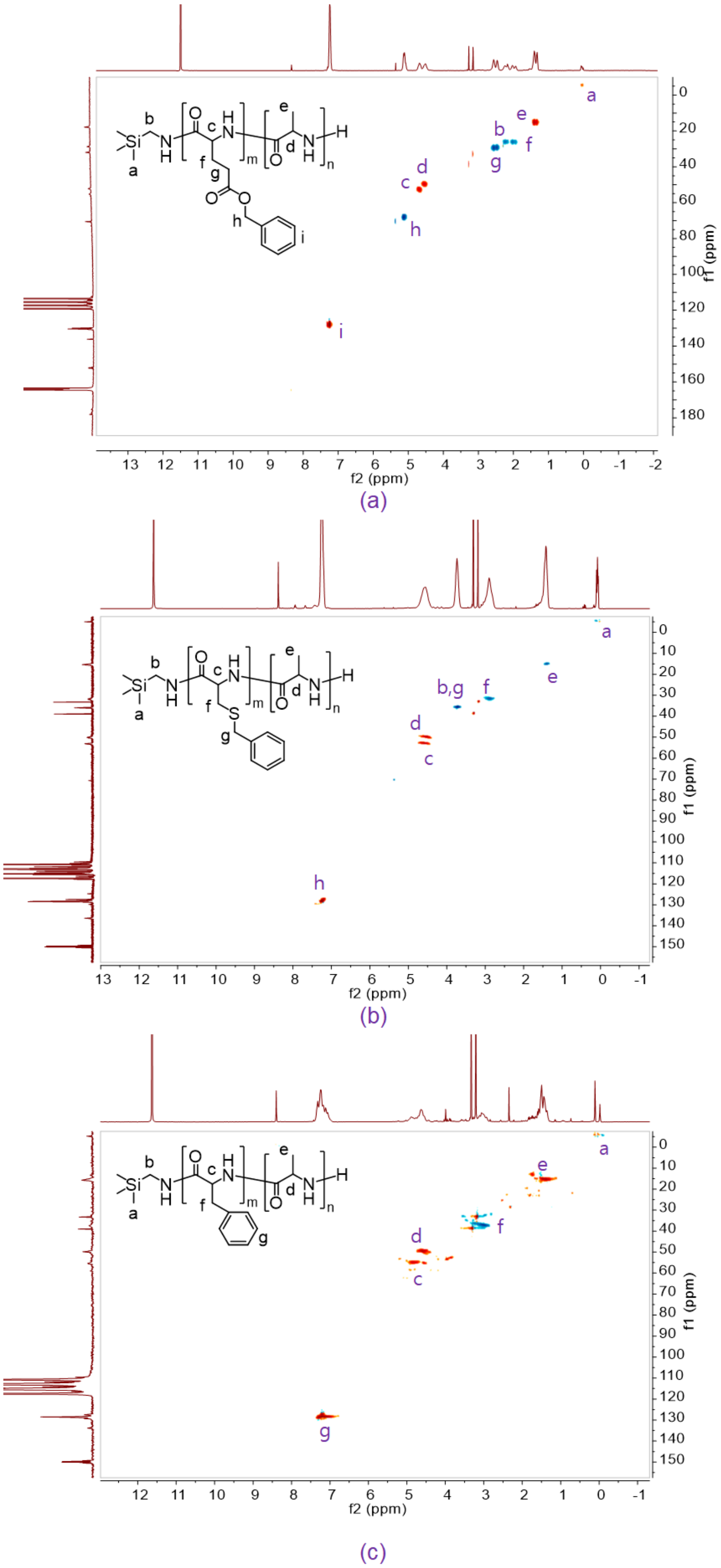

Figure S2. HSQC spectra of (**a**) poly(Ala-*co*-Bn-Glu), (**b**) poly(Ala-*co*-Bn-Cys), and (**c**) poly(Ala-*co*-Phe) measured in trifluoroacetic acid (TFA-d).

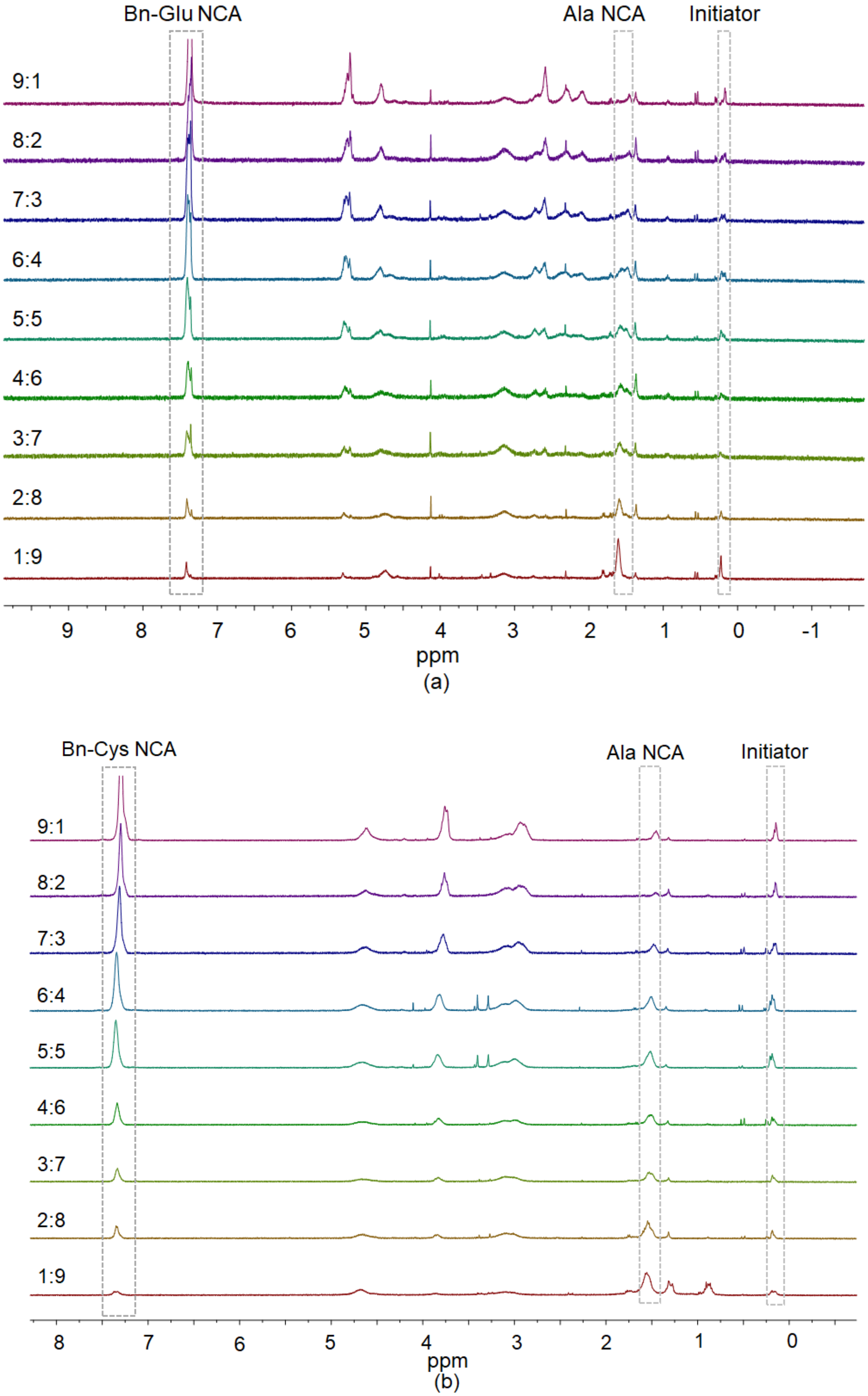
Bn-Glu NCA
Ala NCA
Initiator
9:1
8:2
7:3
6:4
5:5
4:6
3:7
2:8
1:9
9
8
7
6
5
4
3
2
1
0
-1
ppm
(a)
Bn-Cys NCA
Ala NCA
Initiator
9:1
8:2
7:3
6:4
5:5
4:6
3:7
2:8
1:9
8
7
6
5
4
3
2
1
0
ppm
(b)

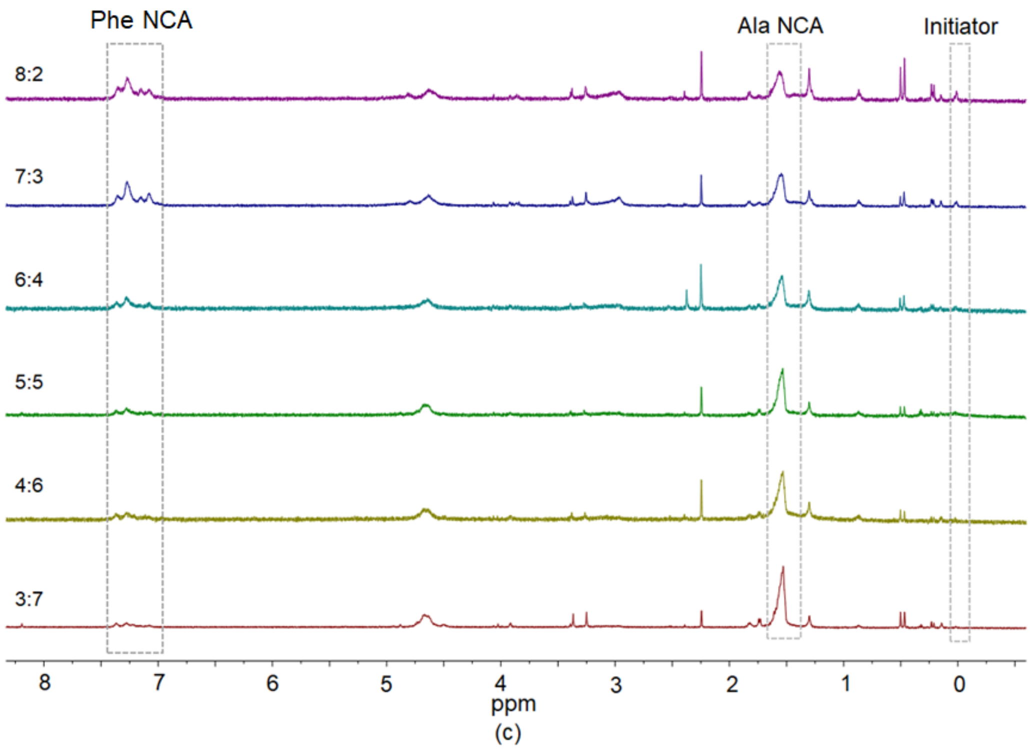

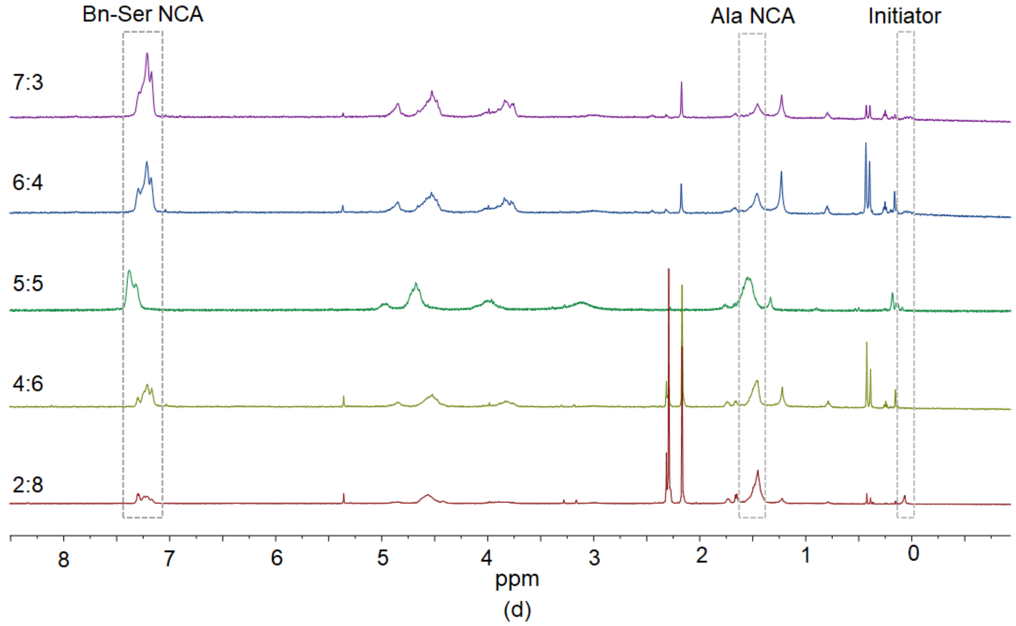

Figure S3. ^{1}H NMR spectra of (**a**) poly(Ala-*co*-Bn-Glu), (**b**) poly(Ala-*co*-Bn-Cys), (**c**) poly(Ala-*co*-Phe), and (**d**) poly(Ala-*co*-Bn-Ser) collected after 5 min of copolymerizations at a wide range of relative composition. Part of the copolymer samples could not be measured due to the low solubility in trifluoroacetic acid (TFA-d).

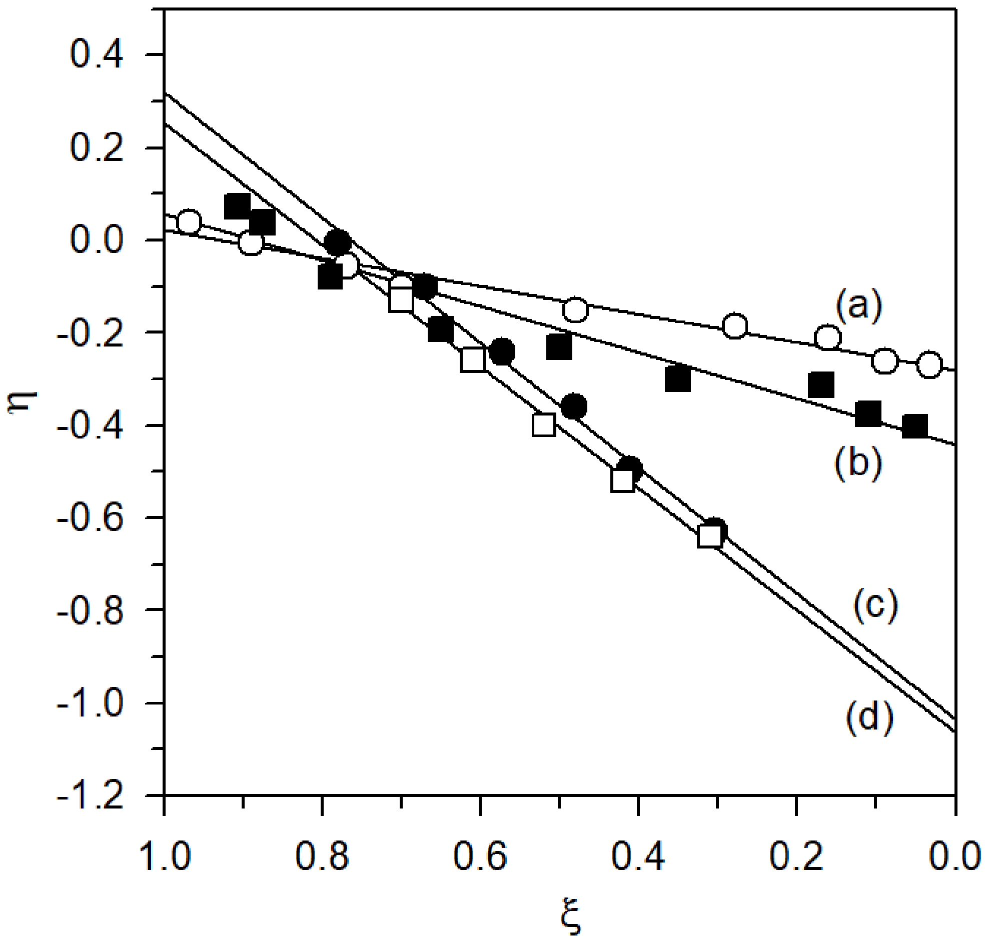

Figure S4. Extended Kelen–Tüdos plots for the determination of the reactivity ratios for (**a**) Bn-Glu NCA and Ala NCA, (**b**) Bn-Cys NCA and Ala NCA, (**c**) Bn-Ser NCA and Ala NCA, and (**d**) Phe NCA and Ala NCA copolymerization systems, where Ala NCA is monomer 2 in all cases.
